# Impairment of oculomotor functions in patients with early to advanced amyotrophic lateral sclerosis

**DOI:** 10.1007/s00415-023-11957-y

**Published:** 2023-09-15

**Authors:** Elisa Aust, Sven-Thomas Graupner, René Günther, Katharina Linse, Markus Joos, Julian Grosskreutz, Johannes Prudlo, Sebastian Pannasch, Andreas Hermann

**Affiliations:** 1 Department of Neurology, Technische Universität Dresden, Dresden, Germany; 2 Verkehrspsychologie, Fakultät Verkehrswissenschaften, Technische Universität Dresden, Dresden, Germany; 3 Deutsches Zentrum für Neurodegenerative Erkrankungen (DZNE) Dresden, Dresden, Germany; 4 Interactive Minds Research, Interactive Minds Dresden GmbH, Dresden, Germany; 5 Precision Neurology and Cluster “Precision Medicine in Inflammation”, University of Lübeck, Lübeck, Germany; 6 Department of Neurology, University of Rostock, Rostock, Germany; 7 Deutsches Zentrum für Neurodegenerative Erkrankungen (DZNE) Rostock/Greifswald, Rostock, Germany; 8 Engineering Psychology and Applied Cognitive Research, Technische Universität Dresden, Dresden, Germany; 9 Center for Transdisciplinary Neurosciences Rostock (CTNR), University Medical Center Rostock, University of Rostock, Rostock, Germany; 10 Translational Neurodegeneration Section “Albrecht Kossel”, Department of Neurology, University of Rostock, Rostock, Germany

**Keywords:** Amyotrophic lateral sclerosis, Eye movements, Oculomotor function, Eye tracking communication systems, Executive function, Saccades

## Abstract

Amyotrophic lateral sclerosis (ALS) can result into an incomplete locked in state (iLIS), in which communication depends on eye tracking computer devices. Oculomotor function impairments in ALS have been reported, but there is little research, particularly with respect to patients in iLIS. In the present study, we compared reflexive and executive oculomotor function by means of an eye tracking test battery between three groups: advanced ALS patients in iLIS (n = 22), patients in early to middle ALS stages (n = 44) and healthy subjects (n = 32). Patients with ALS showed significant deteriorations in oculomotor functions, with stronger impairments in iLIS. More specifically, ALS patients produced visually guided prosaccades with longer latencies and more frequent hypometria compared to healthy subjects. Longest latencies were obtained in iLIS patients, with a stronger prolongation for vertical than for horizontal prosaccades. ALS patients made more antisaccade errors and generated antisaccades with longer latencies. Smooth pursuit was also impaired in ALS. In the earlier ALS stages, bulbar onset patients presented stronger antisaccade and smooth pursuit deficits than spinal onset patients. Our findings reveal a relevant deterioration of important oculomotor functions in ALS, which increases in iLIS. It includes impairments of reflexive eye movements to loss of executive inhibitory control, indicating a progressing pathological involvement of prefrontal, midbrain and brainstem areas. The assessment of oculomotor functions may therefore provide clinically relevant bio- and progression marker, particularly in advanced ALS.

**Supplementary Information:**

The online version contains supplementary material available at 10.1007/s00415-023-11957-y.

## Introduction

Amyotrophic lateral sclerosis (ALS) is the most prevalent and a fatal degenerative motoneuron disease. Due to progressive muscle weakness and loss of muscle control [1, 2], ALS can lead to an incomplete locked in state (iLIS). Patients in iLIS are tetraplegic. They are immobile and unable to speak, but remain fully conscious and can control their eye movements [3, 4]. Active and complex communication therefore relies exclusively on eye tracking communication systems (ETCS), which is crucial especially in advanced ALS for maintaining patients’ quality of life and even their will to live [5–7].

Due to the resistance of oculomotor nuclei’s to ALS pathophysiology [8], it was assumed that eye movement control is relatively spared [9–11]. However, it has been shown that oculomotor functioning can be affected in ALS, primarily due to the pathological processes in non-motor brain regions [10–12].

Eye movement control relies on well-defined neural networks [12–14] and may therefore reflect alterations of the central nervous system [15]. Eye tracking technology enables the precise measurement of eye movements in ALS up to iLIS [10]. Investigating oculomotor dysfunctions can therefore expand our knowledge about neurodegenerative processes and pathophysiological mechanisms in ALS disease [12, 15]. Accordingly, oculomotor changes in ALS can potentially serve as much-needed biomarker throughout the whole disease course [10], thereby contributing to diagnostics, prognostics and monitoring [16, 17]. This is particularly relevant in iLIS, when other voluntary motor functions are lost.

Deteriorations in oculomotor function can also indicate impairment of cognitive, including executive functions [10, 18]. This is important because cognitive deficits are common, non-motor symptoms in ALS [19–21], but cannot be (reliably) identified by standard neuropsychological tests in advanced stages, at the latest in iLIS. Focusing on advanced ALS, reduced eye movement control can hinder the use of ETCS [5, 6] and therefore endanger the patients’ quality of life, safety and self-determination, including end-of-life-decisions. Some iLIS-patients progress into a complete locked in state with a total loss of voluntary eye movement control [3, 22].

Research so far has examined changes in oculomotor control in early to middle ALS stages. Impairments in smooth pursuit eye movements have been reported [23–25], whereas visually guided or reflexive saccades (henceforth prosaccades) were often not altered [18, 23, 26, 27]. However, prosaccades were slower in ALS patients with bulbar onset [10, 23] and only in the vertical direction for ALS patients with frontotemporal dementia [28].

Impaired antisaccade performance has been reported at very early stages of ALS [29] and for asymptomatic carriers of the ALS-associated *SOD1* gene [26]. Further evidence revealed longer latencies [18, 23] and more errors in antisaccade tasks [12, 18, 29–31], indicating reduced inhibitory control (i.e. deficits in higher-level oculomotor function) [18].

A sequential classification of oculomotor deficits in ALS has been suggested [29] and considered to be in line with progression of phosphorylated TAR DNA-binding protein (pTDP-43) pathology from frontal cortex into brainstem areas [32]: antisaccade deficits occur early in the disease course (“stage 1”), followed by additional smooth pursuit dysfunctions and slowing of prosaccades (“stage 2”) [29]. In turn, a longitudinal study of oculomotor function in ALS revealed no reliable changes over a time course of 20 months [18]. However, those last studies investigated ALS patients only in early to middle disease stages. Oculomotor research in ALS is importantly limited by neglecting patients in advanced stages, including iLIS [18, 23, 29]. Consequently, there is an unmet need to investigate decline of oculomotor and cognitive function decline during the whole ALS course.

Our cross-sectional study will address this research gap. By analyzing lower-level and higher-level oculomotor functions in (i) iLIS-patients, (ii) ALS patients at the early to middle stage (henceforth referred to as “earlier ALS-patients”), and (iii) healthy controls (HC), we aim for a better understanding of oculomotor impairments over the course of the disease.

We hypothesized a decreasing performance in prosaccade, antisaccade and smooth pursuit tasks from earlier ALS to iLIS patients; HC served as baseline, where best performance for all tasks was predicted. Based on initial indications of a stronger vulnerability of vertical eye movements in ALS [28], we furthermore tested for possible differences in saccadic latencies for vertical vs. horizontal eye movements. Exploratory analyses were performed to compare oculomotor functions between ALS patients with bulbar versus spinal onset, since stronger impairments for bulbar onset were reported [10, 18, 23, 33]. In addition, we explored the progress of oculomotor changes in association with the duration of ALS and the progression of motor deficits.

## Methods

### Sample recruitment

Patients for the earlier ALS group were recruited from the outpatient clinic at the University Hospital in Dresden. Patients for the iLIS group were recruited from specialist outpatient clinics at the University Hospital in Dresden, the Charité Berlin, the University Hospitals in Rostock, Jena, Goettingen, Hannover, and from a patient network (ALS mobil e.V.). The two groups have been recruited sequentially (first iLIS, second earlier ALS) as convenience samples.

For all patients, inclusion criteria were an established diagnosis of ALS or an ALS variant excluding PLS [34]. For iLIS patients, the presence of iLIS as an inclusion criterion was defined as tetraplegia and functional anarthria with loss of mobility and at most minimal residual head or limb movement (e.g. of toes, individual fingers, face muscles). The inclusion criteria of sufficient eye movement control for ETCS use was initially screened on the basis of the patient’s own report or that of their next of kin or attending physician. The successful calibration of the ETCS-system was the second precondition for the study participation. For all patients, exclusion criteria comprised a clinically defined frontotemporal dementia or obvious severe cognitive impairment, evaluated by a psychologist or neurologist with significant experience with ALS and iLIS patients.

In the HC group, we included age-, gender- and education-matched subjects without history of neurological or psychiatric diseases. Patients or control subjects were excluded in case of a known severe horizontal or vertical gaze palsy or other severe oculomotor disturbances that impeded the use of an eye tracking device.

### Sample characteristics

**Fig. 1:**
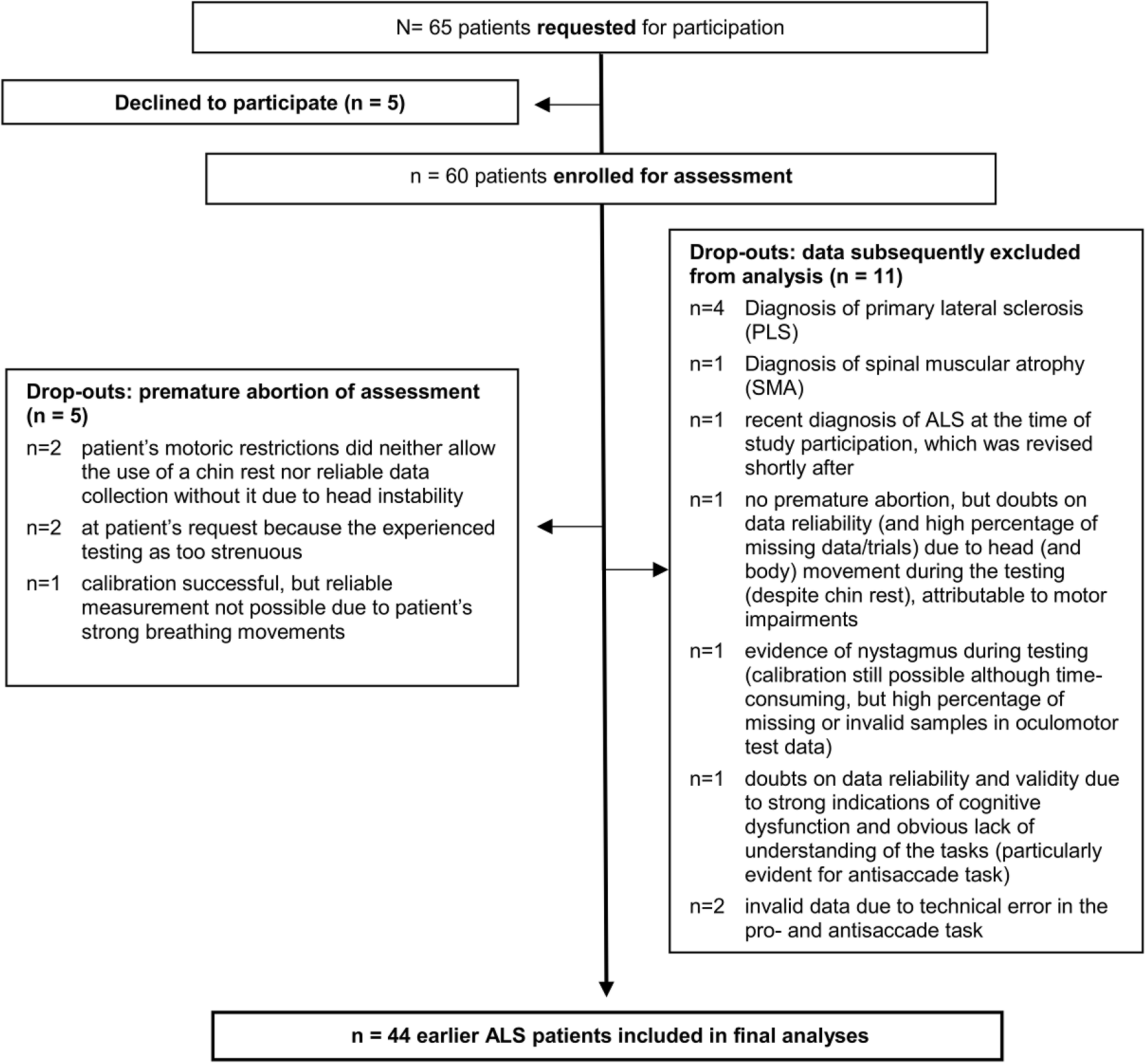
Flowchart of patient sample recruitment and assessment: earlier ALS group

Sixty-five patients in early to middle stage ALS (who were able to be tested in sitting position; ALSFRS ≥ 18) were invited for participation. Five of them declined to participate and 16 were categorized as drop-outs (for details see Fig. 1). Therefore, 44 earlier ALS patients were included in the analyses.

**Fig. 2:**
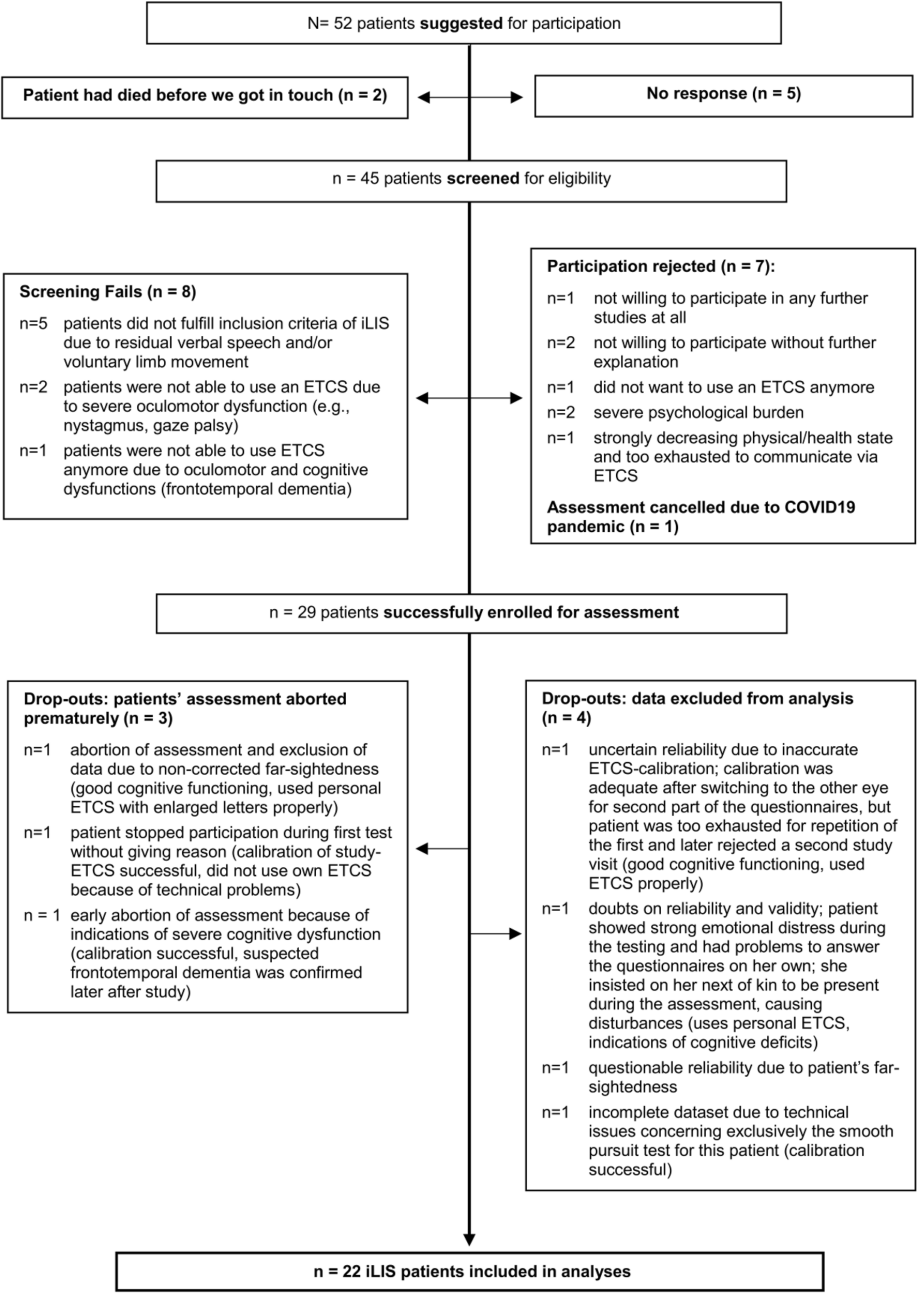
Flowchart of patient sample recruitment and assessment: iLIS group

Fifty-two patients were suggested for participation in the iLIS group, of whom 45 were screened for eligibility. Twenty-eight of them were enrolled in the study, of which 22 iLIS patients were included in the analyses (for details see Fig. 2). In this group, 19 patients used invasive ventilation and all were supplied with a personal ETCS (from different manufactures).

In addition, 34 HC were recruited. One participant had to be excluded due to difficulties in calibration, very likely due to intraocular lenses as treatment of catarcat. Another participant was excluded due to strong indications for the evidence of a psychiatric disorder. Thus, 32 HC were included in the analyses.

### Eye tracking device and data collection procedure

Eye movements were recorded using a monocular Eyegaze Edge^®^ remote infrared ETCS with a sampling rate of 50 Hz (LC Technologies). The ETCS-screen (15.4 inch, 16:9 aspect ratio) was positioned fronto-parallel in a distance of 55–60 cm to the face. An observer screen served to control for a reliable gaze control of the ETCS and to ensure that subjects read the instructions and completed the tests.

The testing procedure differed between the three groups. All iLIS patients were assessed at home or their current nursing home in a light illuminated room. They completed the eye tracking test battery either lying in bed or sitting in a wheelchair.

Earlier ALS patients and HCs were tested at the University Hospital Dresden or in the Technische Universität Dresden in a light illuminated laboratory room. They completed the test battery seated on a chair, with their head rested upon an adjustable chin rest. Earlier ALS patients who were motorically unable to bend towards the chin rest where tested without it, under the condition of minimal head movement to ensure reliable recording of eye movements.

Sociodemographic and disease or health related information were requested directly from the subject, obtained from the patient file or in case of iLIS patients requested from their next of kin or professional caregiver. Disease severity was quantified by the degree of motor impairment using the ALS Functional Rating Scale-Revised (ALSFRS-R) [35]. The scale consists of 12 items and results in a single score with a range from 0 (complete loss of movement, anarthria and dependence of invasive ventilation and invasive nutrition) to 48 (normal motor abilities). Delta ALSFRS-R as a measure of the rate of disease progression was calculated by subtracting the current ALSFRS-score from 48 and dividing it by the ALS duration in months.

All iLIS patients and part of the HC completed the oculomotor function tests as part of a larger eye tracking battery of tests and questionnaires (e.g. on quality of life), for which results have been published [36]. Importantly, the assessment always began with the oculomotor tasks that are subject of this report.

### Oculomotor function measures

The oculomotor tasks were designed, implemented and executed using the software NYAN 3^®^ (Interactive Minds GmbH). Stimuli were presented against a dark-grey background (RGB: 62, 62, 62). The protocol started with a 9-point calibration. Parameters for the automatic calibration ensured an accuracy of at least 0.5°. The subsequent assessment of the oculomotor performance began with the smooth pursuit task, followed by the prosaccade and the antisaccade tasks.

The *prosaccade task* consisted of two blocks (horizontal, vertical) with 48 trials each. The task always began with the horizontal block comprising 24 trials to the left/right, followed by the vertical block with 24 down/up trials. Subjects were instructed to perform a saccade to an appearing target (red dot) as quick and accurately as possible. Each trial began with the presentation of a central white fixation cross (size: 0.8°) and two white anchor dots (size: 0.54°) located at the possible positions of the target stimuli. The dots were located in 9° distance (for 55 cm distance from the screen) from the fixation cross (left and right or above and below). The fixation cross and the anchor dots were replaced (after a randomly varied duration of 1700 ± 800 ms) by a red target point (size: 0.54°), which appeared at the position of one of the two anchor points in quasirandomized order.

The design of the *antisaccade task* (horizontal and vertical block) was identically to the prosaccade task, thus consisting of the same stimuli with the identical temporal and spatial characteristics. In the antisaccade task though, subjects were instructed to execute a saccade in the opposite direction of the red dot (i.e. to the left of the fixation cross when the red dot appeared to the right). All subjects started with the horizontal antisaccades followed by the vertical antisaccades block.

The *smooth pursuit task* consisted of 48 trials (one block), in which subjects were requested to follow the horizontal or vertical movement of a white dot (size: 0.54°) as accurately as possible. The movement originated in quasirandomized order at one of four starting points (with same frequency in total), each located in 9° distance from the center of the screen. Each of the trials started with the static presentation of the dot (for varying duration: 1250 ± 250 ms). It was followed by a sinusoidal movement at *f* = 0.58 Hz to the position of the opposite starting point (turning point) and from there back to the starting point (maximum velocity 30°/s, reached half way through in each motion segment).

### Analysis of oculomotor function

The obtained eye movement data was exported from NYAN 3^®^ and then processed and analyzed using R 4.2.1 [37]. Occurrence of saccades in all three oculomotor tasks was determined by a velocity criterion. For the prosaccade and antisaccade tasks, the start of a saccade was identified in the data by the time of the first sample of a stream of adjacent samples with a sample-to-sample velocity larger than 30°/s. For the smooth pursuit task, the start of a saccade was identified by the threshold of a sample velocity that exceeded the smooth stimulus velocity by 30°/s at a given time, and the end of the saccade by a velocity falling below that threshold.

For the prosaccade and antisaccade task analysis, trials were considered as valid when (i) there were at least 50% of valid samples following the target onset (excluding measurement errors or blinks) and (ii) if the starting gaze position was within 2° of the center of the fixation cross when the target appeared.

Performance in the prosaccade task was evaluated by *latency* and *amplitude* for valid trials with a correct response. Responses were classified as correct when a primary saccade was made in the direction of the target with a latency greater than 180 ms. Latency was calculated as the time between target onset and primary saccade onset [18, 23, 26, 29]. Amplitude was determined as the distance between the subject’s gaze position in the last sample before the target onset and the eye position at the end of the saccade.

Similarly, for the antisaccade task, we analyzed antisaccadic latency and errors for valid trials. Antisaccade latency was determined in the same way as for the prosaccades, but a correct response was defined as a primary saccade in the opposite direction of the red dot. Errors were defined as incorrect reactions in valid trials, so either (i) a primary saccadic response within 180 ms after target onset, (ii) a missing saccadic response or (iii) a primary saccadic response directed toward the target (i.e. a prosaccade) [18, 26, 29, 30]. Error rates represent the ratio between incorrect responses and valid trials (range 0–1). For all patients with error rates > 0.9 in the antisaccade task, we ensured that they corrected any of their errors (i.e. made an antisaccade after the primary prosaccade) to make sure that they had understood the task instruction.

Performance in the smooth pursuit task was evaluated by analyzing *gain* and the number of *catch-up-saccades* for valid trials. Validity of data for motion segments in each trial was determined by a visual inspection, removing trials with large amounts of missing data (i.e. eye blinks or measurement errors), noisy data or non-compliant task behavior. For the gain, only the middle 50% of the trial samples in each motion segment were used to determine the parameter. We excluded samples that were classified as saccades as well as samples adjacent to those saccade samples. Gain was then calculated as the ratio between smooth eye velocity and target velocity [15, 23, 26, 29]. Catch-up-saccades were defined as saccades towards the target and with a final position that did not exceed the current position of the stimulus.

### Statistical analysis

Comparisons of demographic and clinical characteristics between groups were conducted by means of Kruskal–Wallis-test, Chi-square test or Mann–Whitney U test, respectively.

We used marginal models to test for differences in the oculomotor parameters between the three groups. These marginal models are extensions of generalized linear models that require only the mean model of the dependent variable to be correctly specified in terms of the covariates. Distributional assumptions on the dependent variable are not required and the regression parameters in such semi-parametric models can be estimated via so-called generalized estimating equations (GEE). GEE yield consistent estimators of the regression parameters of interest, even if the within-subject associations among the repeated measures have been misspecified. They also provide valid standard errors that correct for misspecifications in the within-subject correlations and for potential over- or underdispersion [38]. Models were specified with an exchangeable working correlation structure, which determines an equal correlation between all pairs of observations within each subject while observations between subjects are uncorrelated. We used a robust covariance estimator that was specifically developed to achieve close to nominal type I error rates for small sample sizes [39]. GEE were conducted using the R package glmtoolbox (version 0.1.3) and the difference of estimated marginal means (DEMM) or odds ratios (OR) were obtained for all models using the R package EMMeans (version 1.9.1-1).

Marginal models for all dependent variables included group (between subject factor, levels: iLIS patients, earlier ALS patients, HC) and direction of the demanded eye movement (within subject factor, levels: horizontal, vertical). We modelled both main effects as well as the interaction between the two factors. Age-specific effects were accounted for by including age (in years) and its interaction with group and direction in all models. All significance tests are based on the DEMM at the mean age. In the model for antisaccade errors, the dependent variable was connected with the covariates via a logit link function. Differences between patients with bulbar and spinal disease onset for each oculomotor parameter were analyzed by means of GEEs as well, separately for iLIS and earlier ALS patients. Those marginal models included the variables subgroup (between subject factor: bulbar, spinal) and direction (within subject factor: horizontal, spinal) as well as age to partial out its association with the dependent variables. All post-hoc contrasts were corrected for multiple comparisons using the Holm-Bonferroni method.

Kendall's τ_b_ correlation coefficients were calculated to determine the relationship between ALS duration (months since diagnosis), ALSFRS-R score and Delta ALSFRS-R  with the oculomotor parameters, separately for the two patient groups. For correlation analyses, the mean for each direction (horizontal, vertical) was calculated using the medians for the two respective conditions (right/left or up/down) of each of the oculomotor for each subject. The significance level for all statistical tests was set at α = 0.05.

## Results

### Demographic and clinical sample characteristics

Results are reported for the final sample of N = 98 participants. Table 1 displays its demographic and clinical characteristics. No significant group differences for age and gender proportion between the three groups were found. The proportions of ALS onset variants (bulbar vs spinal) did not differ between earlier ALS and iLIS patients. As expected, iLIS patients presented a significantly longer ALS disease-duration and a lower ALSFRS-R score than earlier ALS patients.

**Table 1:** Demographic and clinical characteristics for all participants included in the analyses

Characteristic	HC subjects	Earlier ALS patients	iLIS patients	*p* value
n	32	44	22	–
Gender, female:male, %	45.5:54.5	54.5:45.5	40.9:59.1	0.53^b^
Age, years^a^	57.0 ± 9.7	60.5 ± 11.2	55.6 ± 7.6	0.19^c^
ALS onset, bulbar:spinal, %	–	29.5:70.5	31.8:68.2	1.0^c^
ALS duration, months^a^	–	28.7 ± 31.0	80.1 ± 58.7	** < 0.001**^d^
ALSFRS-R^a^	–	36.4 ± 7.6	2.8 ± 4.2	** < 0.001**^d^

Statistically significant results written in bold (α = 5%)

*ALS* amyotrophic lateral sclerosis, *ALSFRS-R* ALS Functional Rating Scale Revised, *ETCS* eye tracking computer system, *HC* healthy controls, *iLIS* incomplete locked in state

^a^Data presented as mean ± SD

^b^Kruskal–Wallis-test (three groups)

^c^Chi-square test (two groups)

^d^Mann–Whitney U-test (two groups)

Descriptive statistics for reflexive and executive oculomotor parameters and movement direction can be found in Table S.1 (supplement). The following sections outline the statistical results of the group comparisons by means of the marginal models for all the oculomotor parameters.

### Prosaccades

**Fig. 3:**
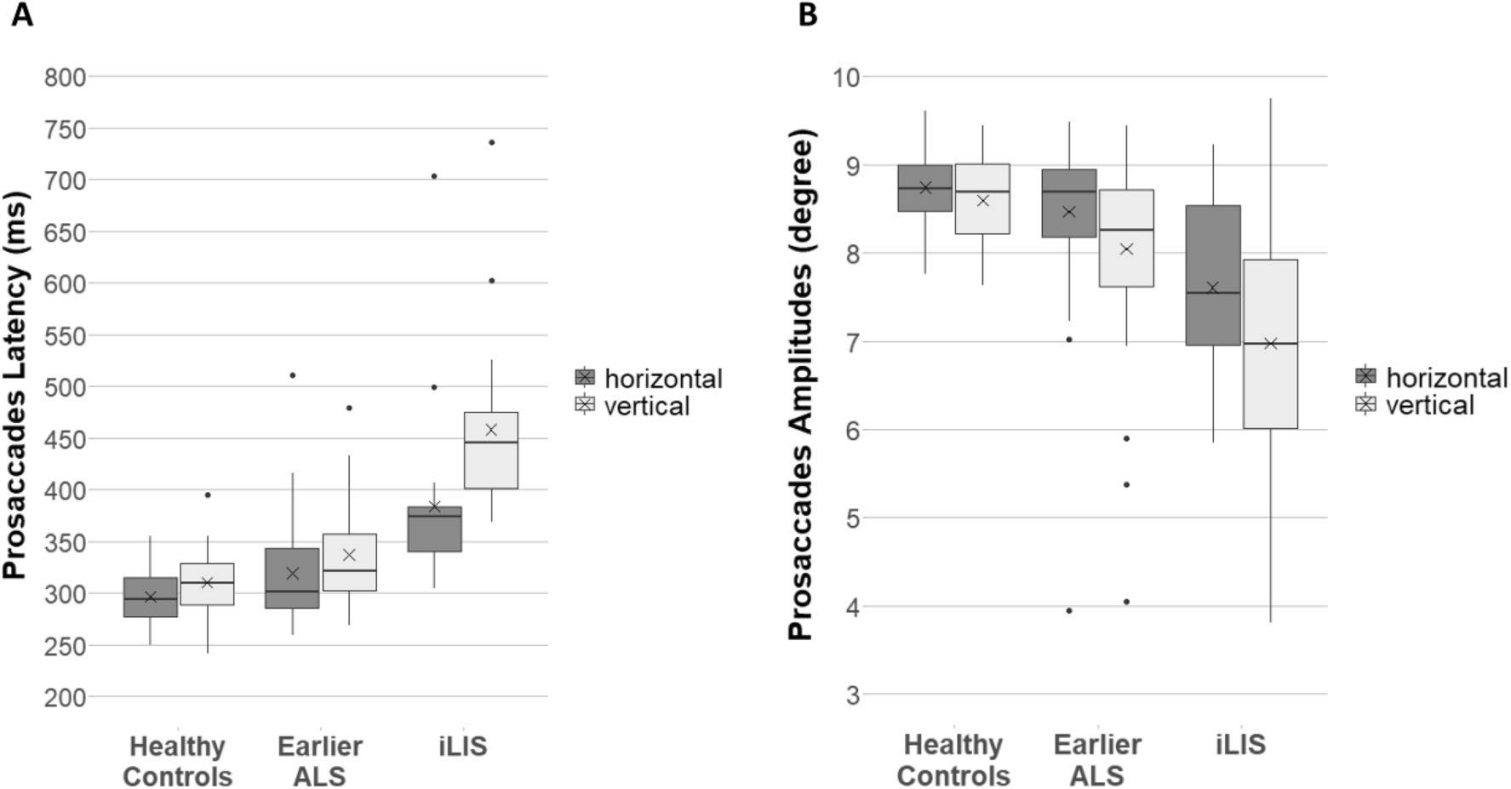
Prosaccade latencies (**A**) and amplitudes (**B**). Boxplots showing the medians (horizontal lines), the means (crosses), the interquartile range (length of the box), the maximum and minimum score excluding outliers (whiskers) and the outliers (dots)

In the prosaccade task, 82.6% of all trials were defined as valid. Examining the *prosaccade latency* with group and direction as independent variables using GEE revealed main effects of group (χ^2^(2) = 89.48, *p* < 0.001) and direction (χ^2^(1) = 34.82, *p* < 0.001) as well as a group by direction interaction (χ^2^(2) = 13.68, *p* = 0.001; see Fig. 3A).

Table 2 displays the results for the pairwise comparisons including confidence intervals. Prosaccade latencies were significantly longer in both directions (horizontal and vertical) in iLIS patients compared to HC and earlier ALS patients. Latencies were also significantly longer in earlier ALS patients compared to HC for both horizontal and vertical prosaccades.

**Table 2:** Inferential statistics results for all pairwise group comparisons using marginal models

Oculomotor parameter	Group comparison	DEMM or OR	CI	*z* value	*p* value
Horizontal prosaccade latency in ms	iLIS vs. HC	89.6^a^	[53.0, 126.2]	6.46	** < 0.001**
	iLIS vs. earlier ALS	71.1^a^	[33.9, 108.2]	5.05	** < 0.001**
	Earlier ALS vs. HC	18.5^a^	[− 4.4, 41.5]	2.13	**0.03**
Vertical prosaccade latency in ms	iLIS vs. HC	146.6^a^	[105.8, 187.5]	9.47	** < 0.001**
	iLIS vs. earlier ALS	121.5^a^	[81.4, 161.6]	7.99	** < 0.001**
	Earlier ALS vs. HC	25.1^a^	[2.34, 48.0]	2.91	**0.007**
Horizontal prosaccade amplitude in degrees	iLIS vs. HC	− 1.33^a^	[− 2.06, − 0.59]	− 4.80	** < 0.001**
	iLIS vs. earlier ALS	− 0.98^a^	[− 1.75, − 0.22]	− 3.39	**0.003**
	Earlier ALS vs. HC	− 0.35^a^	[− 0.72, 0.03]	− 2.41	**0.016**
Vertical prosaccade amplitude in degrees	iLIS vs. HC	− 1.59^a^	[− 2.61, − 0.58]	− 4.13	** < 0.001**
	iLIS vs. earlier ALS	− 1.15^a^	[− 2.21, − 0.098]	− 2.89	**0.012**
	Earlier ALS vs. HC	− 0.44^a^	[− 0.88, − 0.006]	− 2.67	**0.015**
Horizontal antisaccade latency in ms	iLIS vs. HC	213.4^a^	[123.70, 303.20]	6.27	** < 0.001**
	iLIS vs. earlier ALS	161.2^a^	[67.1, 255.20]	4.52	** < 0.001**
	Earlier ALS vs. HC	52.3^a^	[8.65, 95.90]	3.16	**0.003**
Vertical antisaccade latency in ms	iLIS vs. HC	223.5^a^	[146.10, 300.85]	7.62	** < 0.001**
	iLIS vs. earlier ALS	172.4^a^	[91.10, 253.70]	5.59	** < 0.001**
	Earlier ALS vs. HC	51.1^a^	[6.59, 95.50]	3.03	**0.003**
Horizontal antisaccade error rate in %	iLIS vs. HC	6.66^b^	[2.87, 15.44]	5.95	** < 0.001**
	iLIS vs. earlier ALS	3.22^b^	[1.34, 7.74]	3.52	**0.002**
	Earlier ALS vs. HC	2.07^b^	[1.09, 3.92]	3.00	**0.008**
Vertical antisaccade error rate in %	iLIS vs. HC	2.82^b^	[1.57, 5.05]	4.69	** < 0.001**
	iLIS vs. earlier ALS	2.00^b^	[0.97, 4.14]	2.52	**0.02**
	Earlier ALS vs. HC	1.41^b^	[0.77, 2.57]	1.49	0.13
Horizontal smooth pursuit gain	iLIS vs. HC	− 0.33^a^	[− 0.44, − 0.22]	− 7.66	** < 0.001**
	iLIS vs. earlier ALS	− 0.17^a^	[− 0.27, − 0.07]	− 4.66	** < 0.001**
	Earlier ALS vs. HC	− 0.16^a^	[− 0.28, − 0.04]	− 3.39	**0.003**
Vertical smooth pursuit gain	iLIS vs. HC	− 0.09^a^	[− 0.18, 0.001]	− 2.62	**0.027**
	iLIS vs. earlier ALS	− 0.0006^a^	[− 0.07, 0.07]	− 0.02	0.98
	Earlier ALS vs. HC	− 0.09^a^	[− 0.18, 0.003]	− 2.54	**0.027**
Horizontal smooth pursuit number of catch up saccades per trial	iLIS vs. HC	1.01^a^	[0.49, 1.53]	5.12	** < 0.001**
	iLIS vs. earlier ALS	0.77^a^	[0.24, 1.29]	3.86	** < 0.001**
	Earlier ALS vs. HC	0.24^a^	[− 0.14, 0.61]	1.68	0.19
Vertical smooth pursuit number of catch up saccades per trial	iLIS vs. HC	0.45^a^	[0.08, 0.83]	3.19	**0.006**
	iLIS vs. earlier LS	0.33^a^	[− 0.04, 0.69]	2.38	0.052
	Earlier ALS vs. HC	0.13^a^	[− 0.17, 0.42]	1.21	0.26

Results are presented for the pairwise comparisons between the respective two groups

P values for statistically significant differences are written in bold (α = 0.05)

*ALS* amyotrophic lateral sclerosis, *CI* confidence interval, *DEMM* difference of estimated marginal means, *HC* healthy controls, *iLIS* incomplete locked in state, *OR* odds ratio

^a^Difference of estimated marginal means (DEMM)

^b^Odds ratio (OR)

Contrast analysis demonstrated stronger latency differences between iLIS patients and HC for vertical prosaccades compared to horizontal prosaccades (DEMM = − 57.0, CI [− 94.06, − 20.0], z = − 3.69, *p* < 0.001). This was also true for latency differences between iLIS patients and earlier ALS patients (DEMM = − 50.4, CI [− 87.58, − 13.3], z = − 3.25, *p* = 0.002). No reliable differences were found between horizontal and vertical prosaccade latencies for the group comparison of ALS patients and HC (DEMM = − 6.6, CI [− 21.8, 8.61], z = − 1.04, *p* = 0.30).

The model for *prosaccade amplitudes* showed only a main effect of group (χ^2^(2) = 42.11, *p* < 0.001) but not of direction (χ^2^(1) = 3.71, *p* = 0.054) and no interaction (χ^2^(2) = 0.58, *p* = 0.75); see Fig. 3B).

Pairwise comparisons (Table 2) showed differences between all groups: amplitudes were shorter for iLIS patients compared to HC and earlier ALS patients for both horizontal and vertical prosaccades. Earlier ALS patients showed also significantly shorter horizontal and vertical prosaccade amplitudes than HC.

Contrast analysis revealed no differences between horizontal and vertical prosaccade amplitudes for comparisons between the groups: iLIS patients compared with HC (DEMM = 0.27, CI [− 0.87, 1.41], z = 0.57, *p* = 1.0), iLIS patients compared with earlier ALS patients (DEMM = 0.17, CI [− 0.99, 1.34], z = 0.35, *p* = 1.0), and earlier ALS patients compared with HC (DEMM = 0.098, CI [− 0.31, 0.51], z = 0.57, *p* = 1.0).

### Antisaccades

In the antisaccade task, 83.6% of all trials were defined as valid.

**Fig. 4:**
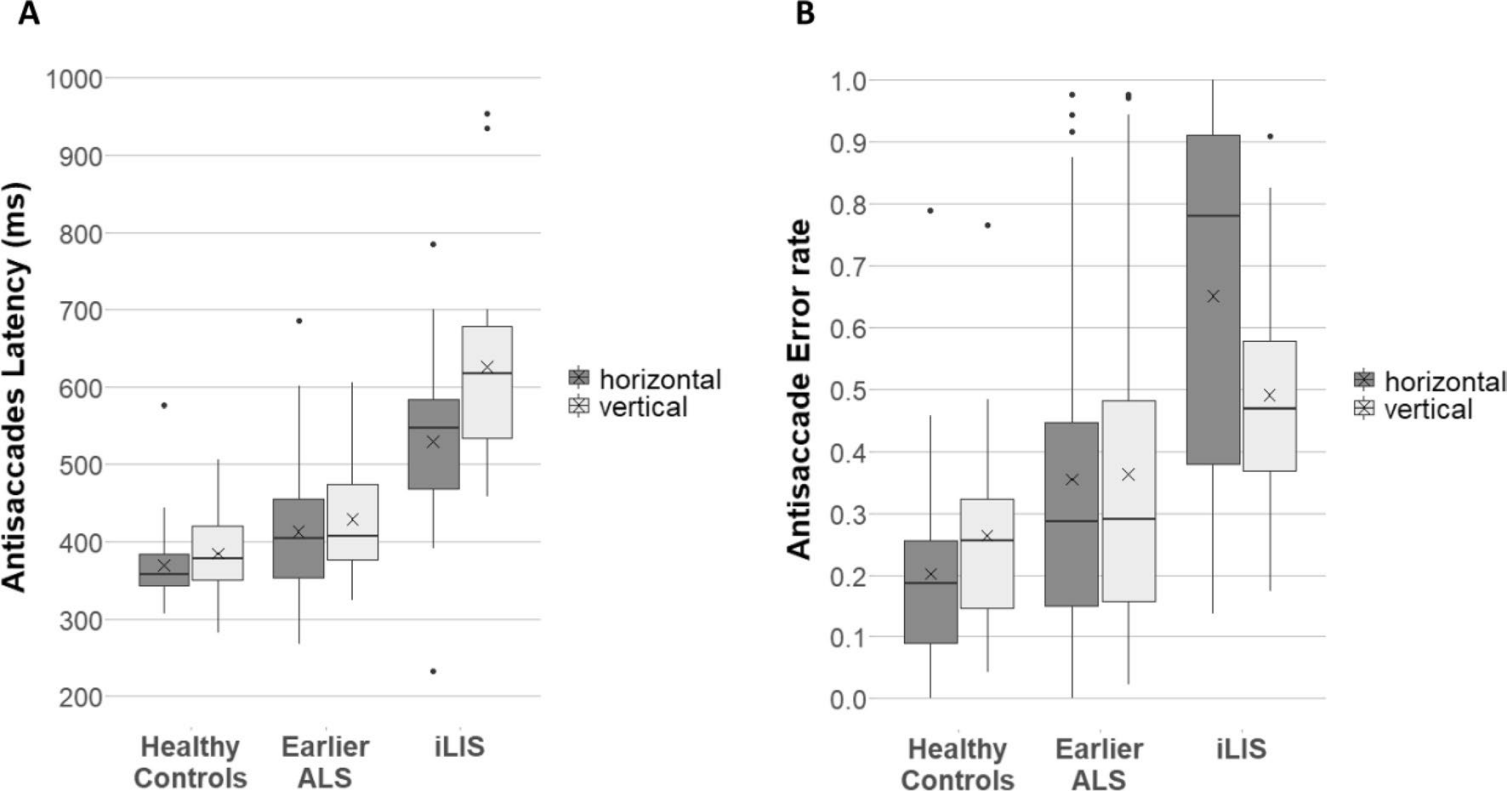
Antisaccade latencies (**A**) and error rates (**B**). Boxplots showing the medians (horizontal lines), the means (crosses), the interquartile range (length of the box), the maximum and minimum score excluding outliers (whiskers) and the outliers (dots)

The model for *antisaccade latencies* showed main effects of group (χ^2^(2) = 60.68, *p* < 0.001) and direction (χ^2^(1) = 10.13, *p* = 0.002). No interaction effect was observed (χ^2^(2) = 0.19, *p* = 0.91; see Fig. 4A).

Pairwise comparisons (Table 2) revealed differences between all three groups: significantly longer antisaccade latencies were found for iLIS patients compared with HC and with earlier ALS patients, whereas earlier ALS patients showed longer antisaccade latencies than HC (all for horizontal and vertical).

Contrast analysis revealed no differences between horizontal and vertical antisaccade latencies for comparisons between the three groups, neither for iLIS patients compared to HC (DEMM = − 10.04, CI [− 71.0, 50.9], z = − 0.39, *p* = 1.0), nor for iLIS patients compared to earlier ALS patients (DEMM = − 11.27, CI [− 73.2, 50.7], z = − 0.44, *p* = 1.0), nor for earlier ALS patients compared to HC (DEMM = 1.22, CI [− 25.9, 28.3], z = 0.108, *p* = 1.0).

The model for *antisaccade errors* revealed only a main effect of group (χ^2^(2) = 35.44, *p* < 0.001) but not for direction (χ^2^(1) = 1.39, *p* = 0.24). There was an interaction between both factors (χ^2^(2) = 14.94, *p* < 0.001; see Fig. 4B).

Pairwise comparisons (Table 2) revealed that iLIS patients made significantly more errors than HC and also more than earlier ALS patients for antisaccades (horizontal and vertical). Earlier ALS patients showed significantly more errors than HC, but only for horizontal antisaccades.

### Smooth pursuit

In the smooth pursuit task, 95.2% of all trials were defined as valid.

**Fig. 5:**
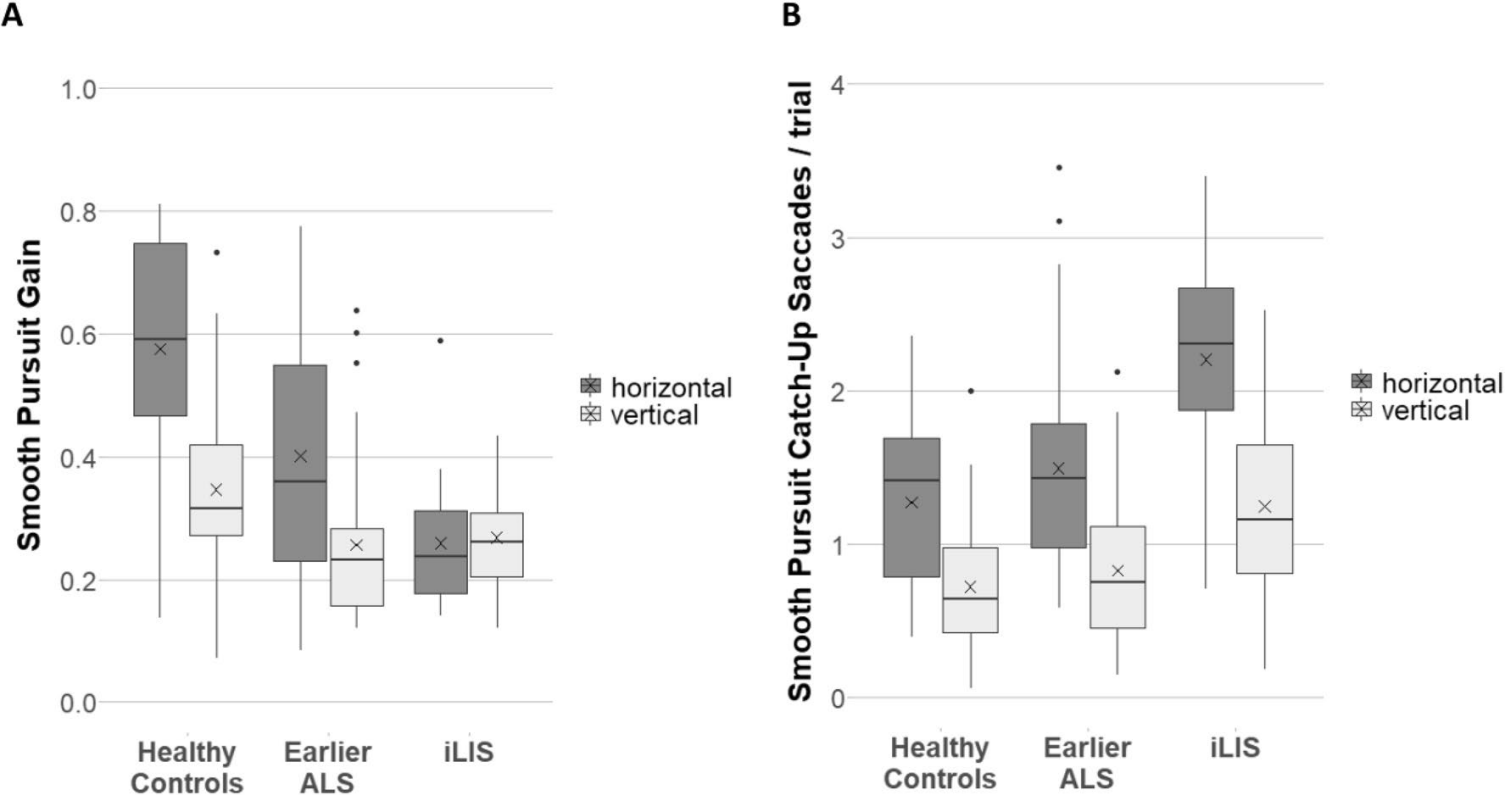
Smooth pursuit gain (**A**) and number of catch-up saccades (**B**). Boxplots showing the medians (horizontal lines), the means (crosses), the interquartile range (length of the box), the maximum and minimum score excluding outliers (whiskers) and the outliers (dots)

The model for *smooth pursuit gain* showed main effects of group (χ^2^(2) = 39.20, *p* < 0.001) and direction (χ^2^(1) = 75.49, *p* < 0.001) as well as an interaction effect between both factors (χ^2^(2) = 48.46, *p* < 0.001; see Fig. 5A).

Pairwise comparisons (Table 2) revealed significant differences between all three groups for gain of horizontal smooth pursuit: iLIS patients presented lower gains compared to HC and earlier ALS patients, while earlier ALS patients demonstrated lower gains than HC.

For *vertical* smooth pursuit, significantly lower gains were found in both patient groups when compared to HC, but no difference was obtained between the patient groups.

The model for the *number of catch-up saccades* per trial in smooth pursuit showed evidence for main effects of group (χ^2^(2) = 23.54, *p* < 0.001) and direction (χ^2^(1) = 174.30, *p* < 0.001) as well as an interaction between the two factors (χ^2^(2) = 11.63, *p* = 0.003; see Fig. 5B).

Pairwise comparisons (Table 2) revealed that iLIS patients presented significantly more catch-up saccades compared to HC in both directions. Compared with earlier ALS patients, iLIS patients showed significantly more horizontal catch-up-saccades, with no differences in vertical catch-up-saccades. There were no differences between earlier ALS patients and HC.

### Associations of oculomotor function with ALS onset type, disease duration and physical impairment

Comparison of patients with bulbar and spinal ALS onset revealed differences in two oculomotor variables, but only in the earlier ALS group: bulbar onset patients compared to spinal onset patients made more errors in the horizontal antisaccade task (OR = 0.81, *p* = 0.034) and showed lower gains in the smooth pursuit task (horizontal: DEMM = 0.15, *p* = 0.006; vertical: DEMM = 0.08, *p* = 0.048), see Table S.2 and S.3 (Supplement) for details.

Only for earlier ALS patients, a longer disease duration was weakly correlated with lower amplitudes of horizontal prosaccades, i.e. more hypometric saccades (τ_b_ = − 0.25, *p* = 0.016). There was also a weak correlation between a lower ALSFRS-R score (i.e. stronger physical impairment) and lower amplitudes of horizontal prosaccades in the earlier ALS group (τ_b_ = − 0.31, *p* = 0.004). A lower ALSFRS-R score was weakly correlated with longer horizontal antisaccade latencies in this group (τ_b_ = − 0.23, *p* = 0.027). No further correlations were found, see Table S.4 (Supplement).

## Discussion

The present study investigated reflexive and executive oculomotor functions in ALS-iLIS patients, ALS patients in earlier disease stages and in HC. There are three main results: Firstly, we found evidence of a deterioration in reflexive and executive oculomotor functions in ALS and over the course of the disease. Secondly, we found that this deterioration is influenced by the ALS onset variants only for horizontal antisaccade errors and smooth pursuit gain only in the earlier ALS subsample. Thirdly, the weak to none correlations between oculomotor functions and ALS duration, ALS disease severity and progression suggest that oculomotor deficits do not simply intensify in parallel to the progression of main motor symptoms.

More specifically, the deterioration in reflexive oculomotor functions was characterized by a prolongation of prosaccade latencies, as well as a significant undershoot (i.e. hypometria) and generally less accurate prosaccades (i.e. dysmetria) in patients in stage of iLIS. These results point to serious changes of reflexive saccadic function in advanced stages of ALS. There was also a slight prolongation in early stage ALS patients compared to HC, while previous work has suggested no impairment of prosaccade latencies in early to middle stages of ALS [18, 23, 26, 27].

The present results, particularly for iLIS patients, support and complement the proposed staging of structural and subsequent functional changes in ALS [29]—which was based on data for earlier ALS only. It suggests that a stronger slowing of prosaccades is characteristic of more advanced ALS-related oculomotor dysfunctions (oculomotor “stage 2”). According to knowledge about prosaccades [12, 13, 23], prolonged latencies can be attributed to TDP-43 pathology spreading into brainstem regions in more advanced ALS stages [12, 29], causing lesions of saccadic burst and omnipause neurons [13].

Regarding explanations for longer latencies for vertical compared to horizontal prosaccades in iLIS patients, burst neurons for horizontal saccades are located in the pons, those for vertical saccades in the midbrain [13, 14]. A post-mortem analysis revealed TDP-43 pathology in brainstem and midbrain in approximately 50% of the ALS patients [40]. A large imaging study evidenced hypermetabolism in midbrain areas in early ALS, construed as correlates of neurodegeneration [41].

The successful generation of antisaccades requires cognitive, particularly inhibitory control [42]. The error rate is the most important parameter of antisaccade performance as it reveals a failure of inhibitory control. Higher error rates for horizontal antisaccades in earlier ALS patients compared to HC confirm previous findings [18, 26, 29, 30, 43] and are also represented in the proposed oculomotor staging model [29]: the isolated increase of antisaccade errors characterize oculomotor “stage 1”. These early deficits are associated with the progression of pTDP-43 pathology into the dorsolateral prefrontal cortex (DL-PFC) [29], which is responsible for the required inhibitory of prosaccades in antisaccade tasks [13, 42, 44, 45]. It was proven that the increased error rate in ALS patients is associated with a reduced activation of the DL-PFC [43]. Consistently, pathological changes of the DL-PFC have been shown to be a structural correlate of impairments in executive functions in ALS [46]. Cognitive deficits affect 30–50% of non-demented early to middle stage ALS patients [19, 21, 47–49], most often the executive functions domain [19, 20, 47, 50, 51].

The increased antisaccade error rate in iLIS patients suggests a relevant deterioration in the course of ALS. This complements and challenges previous longitudinal findings of a stable cognitive function for pre-iLIS ALS patients [18, 52, 53], including antisaccade performance [18]. Antisaccade error rates correlate with performance on more complex neuropsychological tests of executive function control [45]. Hence, our findings support the view that executive dysfunctions develop very early in ALS, but challenge the conclusion drawn from longitudinal studies that such deficits remain stable throughout the disease [53].

Regarding the higher antisaccade error rates in earlier ALS patients with bulbar versus spinal onset, literature is inconsistent: two meta-analyses do not confirm an association between bulbar onset and stronger cognitive or particularly executive dysfunction [19, 20], but a recent one finds bulbar onset to be a strong predictor of cognitive impairment and frontotemporal dementia in ALS [33].

Studies of smooth pursuit in ALS are rare and mostly did not employ eye tracking [9, 10, 23–25]. Some of them did not prove an impaired performance [26, 29], others only in a very small proportion of the patients [25] However, deficits in horizontal smooth pursuit were also characterized as a feature of ALS [10], which is supported by our results. As we found those deficits in earlier ALS patients already but they were more pronounced in iLIS, they indicate a serious progression of oculomotor impairments.

The already mentioned staging model suggests smooth pursuit deficits to appear later in ALS [29]: frequent catch-up-saccades in smooth pursuit—in addition to antisaccade deficits (of “stage 1”)—characterize oculomotor “stage 2”. This transition is explained by the progression of pathological changes from the frontal lobe into the lower brainstem. Our finding of strong impairments in iLIS patients compared to both earlier ALS patients and HC is a supportive addition to this model. However, smooth pursuit movements are generated by a complex network [14, 54, 55]. As suggested by clinical and imaging studies, catch-up-saccades might be caused by dysfunctions of the ponto-cerebellar pathways [12, 13, 54]—while a reduced gain might reflect impaired pursuit movement control by fronto-striatal pathways [29]. Especially the frontal eye fields seem to be responsible for the reduced gain in ALS [12, 55]. On this basis, our findings may support the progression of pathological changes from frontal regions towards brainstem: iLIS patients show both increased catch-up saccades and a reduced gain, whereas earlier ALS patients show only reduced gain compared to HC. Interestingly, Poletti et al. observed that patients with ALS-specific cognitive disturbances in the ECAS were also significantly more likely to show abnormal smooth pursuit and/or prosaccade performance on clinical bedside assessment [25].

We observed less pronounced group differences for *vertical* smooth pursuit. Since higher speed of smooth pursuit degrades performance [55, 56] and vertical pursuit is known to be slower than horizontal in healthy subjects [56], this finding might present a floor effect due to the high velocity of the demanded pursuit movement.

A relevant implication of the oculomotor changes is their prospective negative impact on use of ETCS in iLIS. A good subjective quality of life with ALS was also reported for patients in iLIS [36, 57, 58]. However, it is strongly related to their access to ETCS [5–7, 59], which they use extensively in daily life [36, 60]. Since these systems require intact oculomotor and cognitive functions, any deterioration of these functions might reduce the system’s usability [61]. Problems in ETCS use are frequent, but often detected belatedly and their oculomotor or/and cognitive causes are almost impossible to identify. Our findings support the possibility and relevance of a systematic eye tracking based assessment of such potential reasons. The changes in eye movement coordination and control we observed, e.g. a slowed generation of voluntary saccades, a reduced ability to suppress visually guided eye movements and inaccurate saccades, might substantially reduce the efficiency and/or speed of ETCS use. Based on knowledge about the natural course of oculomotor and neurocognitive functions in ALS, their monitoring could allow to detect changes earlier and more precisely—and consequently to predict a deterioration that might compromise the ability to communicate via ETCS. There are cases of patients merging into a *complete* locked in state [3, 22] and around 10% of ALS patients develop frontotemporal dementia [19]. This means, deficits can lead to a state in which patients cannot reliably *make* and/or *communicate* their will and decisions—including end-of-life decisions. To detect such a progress would allow to prepare for it, e.g. by recording patients’ wishes as long as it is still (reliably) possible.

A main limitations of the present study is its small sample size, which is particularly limiting regarding the heterogeneity of ALS pathology and its clinical presentation [1, 62]. Moreover, a probable selection bias must be considered, especially for the iLIS group (e.g. regarding the willingness to participate in a demanding eye tracking based study, use of personal ETCS). In support of this bias, screening failures and drop-outs were mostly due to severe oculomotor and cognitive deficits. At the same time, those frequent drop-outs emphasize the relevance of oculomotor functioning for ETCS use. Inevitable differences in the test settings between iLIS patients and all other participants limit  comparability between the groups. A possible influence of apathy on, for example, the initiation of eye movements was not examined but must be considered, since apathy is the most prevalent symptom of behavioral impairment in ALS [63].

The patients in our study were not systematically screened for genetic variations, which should be considered in future studies especially including UNC13A snips [64]. We did not subject the patients in our study to (classic) neuropsychological testing (e.g. using the ECAS), which would have been possible only for the earlier ALS and HC group. In future research, a comprehensive eye-tracking based battery of cognitive function could provide differential insights into neurocognitive consequences of ALS, also in these patients with strongly impaired motor functions and thus up to iLIS. This future research should also assess the negative effects of oculomotor changes on ETCS use in order to identify early and sensitive marker of such restraints. Moreover, imaging evidence (PEG, MRI) would be needed to verify hypotheses about neuronal correlates of changes in oculomotor performance. Overall, our results emphasize the need for longitudinal studies with longer follow-up periods than previous ones. Such studies could verify the suggested potential of oculomotor functions as biomarker of pathological processes in advanced ALS stages up to and within iLIS.

Taken together, the presented study evidences relevant and progressing deficits of reflexive and executive oculomotor functions in ALS. Antisaccade error rates might be a valuable, easily assessable measure of executive function and frontal lobe impairment in all ALS stages and can be interpreted without the confounding effects of pure *motor* slowing of gaze. Our findings indicate a deterioration that successively leads to extensive, global dysfunctions in iLIS, which would correspond to the typical concepts of disease spreading in neurodegenerative diseases.

### Supplementary Information

Below is the link to the electronic supplementary material.

Supplementary file1 (DOCX 59 KB)

## Data Availability

Data not provided in the article may be shared (anonymized) at the request of any qualified investigator for purposes of replicating procedures and results.
