# Supplementary material for: Impairment of oculomotor functions in patients with early to advanced amyotrophic lateral sclerosis
Source: J Neurol. 2023 Sep 15;271(1):325–39. doi: 10.1007/s00415-023-11957-y (PMC10770212; doi:10.1007/s00415-023-11957-y)
Supplement: Supplementary file 1 — Supplementary file1 (DOCX 59 KB) [file 415_2023_11957_MOESM1_ESM.docx]

# Supplement

| **Table S.1** Descriptive analysis results for oculomotor test parameters | | | |
| --- | --- | --- | --- |
| **Oculomotor parameter** | **HC subjects** | **Earlier ALS**  **patients** | **iLIS patients** |
| Horizontal Prosaccade Latency in ms^a^ | 296.7 ± 27.7 | 319.0 ± 48.1 | 383.9 ± 81.7 |
| Vertical Prosaccade Latency in ms^a^ | 310.2 ± 33.0 | 337.2 ± 46.2 | 458.4 ± 83.2 |
| Horizontal Prosaccade Amplitude in degrees^a^ | 8.74 ± 0.41 | 8.47 ± 0.89 | 7.61 ± 1.01 |
| Vertical Prosaccade Amplitude in degrees^a^ | 8.59 ± 0.51 | 8.05 ± 1.06 | 6.98 ± 1.36 |
| Horizontal Antisaccade Latency in ms^a^ | 369.6 *±* 52.7 | 433.5 *±* 106.72 | 531.2 *±* 123.19 |
| Vertical Antisaccade Latency in ms^a^ | 385.8 *±* 47.52 | 444.1 *±* 102.01 | 639.5 *±* 139.77 |
| Horizontal Antisaccade Error Rate in % | 20.18 *±* 16.36 | 35.51 *±* 27.07 | 65.08 *±* 29.17 |
| Vertical Antisaccade Error Rate in % | 26.4 *±* 14.1 | 36.29 *±* 28.51 | 49.06 *±* 19.54 |
| Horizontal Smooth Pursuit Gain^a^ | 0.58 *±* 0.19 | 0.40 *±* 0.19 | 0.26 ± 0.10 |
| Vertical Smooth Pursuit Gain^a^ | 0.35 ± 0.15 | 0.26 ± 0.13 | 0.27 ± 0.09 |
| Horizontal Smooth Pursuit number of Catch up saccades per trial^a^ | 1.27 ± 0.54 | 1.49 ± 0.65 | 2.20 ± 0.72 |
| Vertical Smooth Pursuit number of Catch up saccades per trial^a^ | 0.72 ± 0.45 | 0.83 ± 0.49 | 1.25 ± 0.59 |
| ᵃData presented as mean ± standard deviation  Abbreviations: ALS = amyotrophic lateral sclerosis; HC = healthy controls; iLIS = incomplete locked in state | | | |

| **Table S.2** Comparisons between earlier ALS patients with spinal and bulbar ALS onset | | | | | | |
| --- | --- | --- | --- | --- | --- | --- |
| **Oculomotor Parameter** | **Spinal**  (n = 31) | **Bulbar**  (n = 13) | **DEMM or OR** | **CI** | ***z* Value** | ***p* Value** |
| Horizontal Prosaccade Latency in ms^a^ | 319.2 ± 55.91 | 318.5 ± 37.18 | 3.62^b^ | [-29.8, 37.0] | 0.24 | 1.0 |
| Vertical Prosaccade Latency in ms^a^ | 331.3 ± 42.65 | 351.2 ± 52.d82 | -2.89^b^ | [-37.8, 32.0] | -0.19 | 1.0 |
| Horizontal Prosaccade Amplitude in degrees^a^ | 8.56 ± 0.52 | 8.24 ± 1.44 | 0.32^b^ | [-0.60, 1.23] | 0.77 | 0.63 |
| Vertical Prosaccade Amplitude in degrees^a^ | 8.23 ± 1.01 | 7.61 ± 1.10 | 0.36^b^ | [-0.45, 1.17] | 1.00 | 0.63 |
| Horizontal Antisaccade Latency in ms^a^ | 412.5 ± 86.43 | 483.5 ± 135.27 | -40.1^b^ | [-104.0, 23.9] | -1.40 | 0.32 |
| Vertical Antisaccade Latency in ms^a^ | 447.2 ± 110.53 | 436.7 ± 81.64 | -37.6^b^ | [-102.0, 26.6] | -1.31 | 0.32 |
| Horizontal Antisaccade Error Rate in %^a^ | **29.05 ± 25.39** | **50.94 ± 25.46** | **0.814^c^** | **[0.67, 0.98]** | **-2.45** | **0.028** |
| Vertical Antisaccade Error Rate in %^a^ | 29.58 ± 27.19 | 52.32 ± 25.89 | 0.834^c^ | [0.68, 1.03] | -1.93 | 0.054 |
| Horizontal Smooth Pursuit Gain^a^ | **0.45 ± 0.19** | **0.28 ± 0.11** | **0.153^b^** | **[0.05, 0.26]** | **3.15** | **0.003** |
| Vertical Smooth Pursuit Gain^a^ | **0.28 ± 0.14** | **0.19 ± 0.08** | **0.080^b^** | **[-0.007 0.17]** | **2.05** | **0.04** |
| Horizontal Smooth Pursuit number of Catch up saccades per trial^a^ | 1.52 ± 0.67 | 1.43 ± 0.62 | 0.079^b^ | [-0.39, 0.56] | 0.37 | 0.71 |
| Vertical Smooth Pursuit number of Catch up saccades per trial^a^ | 0.89 ± 0.50 | 0.68 ± 0.45 | 0.196^b^ | [‑0.17, 0.56] | 1.20 | 0.46 |
| ᵃ Descriptive statistics results presented as mean ± SD  ^b^ Difference of estimated marginal means (DEMM)  ^c^ Odds Ratio (OR)  Inference statistics results are presented for the pairwise comparisons between bulbar onset and spinal onset patients.  Statistically significant differences are written in **bold** (α = 0.05.)  Abbreviations: ALS = amyotrophic lateral sclerosis; CI = confidence interval; DEMM = difference of estimated marginal means; OR = odds ratio | | | | | | |

| **Table S.3** Comparisons between iLIS patients with spinal and bulbar ALS onset | | | | | | |
| --- | --- | --- | --- | --- | --- | --- |
| **Oculomotor Parameter** | **Spinal (n = 15)** | **Bulbar (n = 7)** | **DEMM or OR** | **CI** | ***z* Value** | ***p* Value** |
| Horizontal Prosaccade Latency in ms^a^ | 385.5 ± 91.97 | 380.4 ± 60.14 | -1.81^b^ | [-89.7, 86.1] | -0.05 | 1.0 |
| Vertical Prosaccade Latency in ms^a^ | 452.3 ± 87.56 | 471.5 ± 77.79 | 0.32^b^ | [-73.2, 73.9] | 0.01 | 1.0 |
| Horizontal Prosaccade Amplitude in degrees^a^ | 7.61 ± 1.04 | 7.59 ± 1.04 | -0.14^b^ | [-1.78, 1.49] | -0.197 | 1.0 |
| Vertical Prosaccade Amplitude in degrees^a^ | 7.07 ± 1.48 | 6.79 ± 1.12 | 0.23^b^ | [-1.87, 2.34] | 0.25 | 1.0 |
| Horizontal Antisaccade Latency in ms^a^ | 530.7 ± 118.92 | 532.5 ± 145.26 | -111.1^b^ | [-529.0, 307.0] | -0.596 | 0.72 |
| Vertical Antisaccade Latency in ms^a^ | 603.44 ± 115.82 | 714.8 ± 165.43 | -92.1^b^ | [-318.0, 134.0] | -0.915 | 0.72 |
| Horizontal Antisaccade Error Rate in %^a^ | 65.40 ± 25.32 | 64.40 ± 38.49 | 1.05^c^ | [0.54, 2.05] | 0.173 | 1.0 |
| Vertical Antisaccade Error Rate in %^a^ | 49.48 ± 17.79 | 48.16 ± 24.41 | 1.03^c^ | [0.81, 1.31] | 0.280 | 1.0 |
| Horizontal Smooth Pursuit Gain^a^ | 0.28 ± 0.11 | 0.21 ± 0.07 | 0.079^b^ | [-0.06, 0.22] | 1.276 | 0.23 |
| Vertical Smooth Pursuit Gain^a^ | 0.25 ± 0.095 | 0.30 ± 0.07 | -0.068^b^ | [-0.16, 0.03] | **-**1.570 | 0.23 |
| Horizontal Smooth Pursuit number of Catch up saccades per trial^a^ | 2.19 ± 0.71 | 2.23 ± 0.79 | 0.238^b^ | [-1.01, 1.49] | 0.427 | 1.0 |
| Vertical Smooth Pursuit number of Catch up saccades per trial^a^ | 1.99 ± 0.68 | 1.35 ± 0.36 | -0.185^b^ | [‑0.89, 0.52] | -0.590 | 1.0 |
| ᵃ Descriptive statistics results presented as Mean ± SD  ^b^ Difference of estimated marginal means (DEMM)  ^c^ Odds Ratio (OR)  Inference statistics results are presented for the pairwise comparisons between bulbar onset and spinal onset patients.  Abbreviations: ALS = amyotrophic lateral sclerosis; ; CI = confidence interval; DEMM = difference of estimated marginal means; iLIS = incomplete locked in state, OR = odds ratio | | | | | | |

| **Table S.4** Correlations of oculomotor parameters with ALS duration, ALSFRS-R score and Delta ALSFRS-R | | | | |
| --- | --- | --- | --- | --- |
| **Oculomotor Parameter** | **ALS-Duration (in months)** | | **ALSFRS-R score** | **Delta ALSFRS-R** |
|  | **Earlier ALS patients** | **iLIS**  **patients** | **Earlier ALS**  **patients** | **Earlier ALS**  **patients** |
| Horizontal Prosaccade Latency in ms | τ_b_ = 0.194  *p* = 0.067 | τ_b_ = -0.024  *p* = 0.879 | τ_b_ = -0.125  *p* = 0.239 | τ_b_ = -0.035  *p* = 0.739 |
| Vertical Prosaccade Latency in ms | τ_b_ = 0.123  *p =* 0.244 | τ_b_ = 0.081  *p* = 0.608 | τ_b_ = -0.039  *p* = 0.715 | τ_b_ = -0.098  *p* = 0.347 |
| Horizontal Prosaccade Amplitude in degrees | **τ_b_ = -0.254**  ***p =* 0.016** | τ_b_ = -0.062  *p =* 0.695 | **τ_b_ = 0.306**  ***p =* 0.004** | τ_b_ = -0.060  *p* = 0.564 |
| Vertical Prosaccade Amplitude in degrees | τ_b_ = -0.087  *p =* 0.411 | τ_b_ = -0.167  *p =* 0.290 | τ_b_ = 0.166  *p =* 0.118 | τ_b_ = -0.075  *p* = 0.473 |
| Horizontal Antisaccade Error Rate | τ_b_ = 0.049  *p* = 0.641 | τ_b_ = 0.129  *p* = 0.415 | τ_b_ = -0.077  *p* = 0.472 | τ_b_ = 0.019  *p* = 0.856 |
| Vertical Antisaccade Error Rate | τ_b_ = -0.087  *p =* 0.412 | τ_b_ = -0.005  *p* = 0.976 | τ_b_ = -0.111  *p* = 0.296 | τ_b_ = 0.156  *p* = 0.137 |
| Horizontal Antisaccade Latency in ms | τ_b_ *=* 0.110  *p* = 0.297 | τ_b_ *=* -0.069  *p* = 0.673 | **τ_b_ = -0.235**  ***p =* 0.027** | τ_b_ = 0.141  *p* = 0.179 |
| Vertical Antisaccade Latency in ms | τ_b_ *=* 0.072  *p* = 0.497 | τ_b_ = 0.215  *p* = 0.174 | τ_b_ = -0.183  *p =* 0.085 | τ_b_ = 0.511  *p* = 0.069 |
| Horizontal Smooth Pursuit Gain | τ_b_ *=* -0.097  *p* = 0.357 | τ_b_ = 0.053  *p* = 0.740 | τ_b_ = 0.173  *p* = 0.105 | τ_b_ = -0.020  *p* = 0.848 |
| Vertical Smooth Pursuit Gain | τ_b_ = -0.076  *p* = 0.472 | τ_b_ = -0.014  *p* = 0.928 | τ_b_ = 0.170  *p* = 0.109 | τ_b_ = -0.022  *p* = 0.832 |
| Horizontal Smooth Pursuit number of Catch up saccades | τ_b_ = -0.046  *p* = 0.663 | τ_b_ = -0.033  *p* = 0.833 | τ_b_ = -0.054  *p* = 0.612 | τ_b_ = 0.077  *p* = 0.460 |
| Vertical Smooth Pursuit number of Catch up saccades | τ_b_ = -0.005  *p* = 0.959 | τ_b_ = 0.229  *p* = 0.147 | τ_b_ = -0.109  *p* = 0.306 | τ_b_ = 0.154  *p* = 0.142 |
| Results for Kendall's tau-b correlation coefficient  Statistically significant results written in **bold** (α=5%)  Correlations with ALSFRS and Delta ALSFRS-R were only analyzed for earlier ALS patients, since the variance of ALSFRS-scores was very low in the iLIS group  Abbreviations: ALS = amyotrophic lateral sclerosis; HC = healthy controls; iLIS = incomplete locked in state | | | | |
